# Supplementary material for: A UMLS-based spell checker for natural language processing in vaccine safety
Source: BMC Med Inform Decis Mak. 2007 Feb 12;7:3. doi: 10.1186/1472-6947-7-3 (PMC1805499; doi:10.1186/1472-6947-7-3)
Supplement: Additional file 13 — RAP application directory. Sets up directory for RAP (RDF application for PHP) source code [file 1472-6947-7-3-S13.gz › rap/doc/phpdoc/default/DC_RES.html]

xml version="1.0" encoding="iso-8859-1"?


Docs For Class DC\_RES


## Class DC\_RES

Description

Description |
Methods (details)

Located in /vocabulary/DC\_RES.php (line 23)

Method Summary

Description |
Methods (details)

void
ABSTRACT\_
()

void
ACCESS\_RIGHTS
()

void
ALTERNATIVE
()

void
AUDIENCE
()

void
AVAILABLE
()

void
BIBLIOGRAPHIC\_CITATION
()

void
BOX
()

void
COLLECTION
()

void
CONFORMS\_TO
()

void
CONTRIBUTOR
()

void
COVERAGE
()

void
CREATED
()

void
CREATOR
()

void
DATASET
()

void
DATE
()

void
DATE\_ACCEPTED
()

void
DATE\_COPYRIGHTED
()

void
DATE\_SUBMITTED
()

void
DCMI\_TYPE
()

void
DESCRIPTION
()

void
EDUCATION\_LEVEL
()

void
EVENT
()

void
EXTENT
()

void
FORMAT
()

void
HAS\_FORMAT
()

void
HAS\_PART
()

void
HAS\_VERSION
()

void
IDENTIFIER
()

void
IMAGE
()

void
IMT
()

void
INTERACTIVE\_RESOURCE
()

void
ISO639\_2
()

void
ISO3166
()

void
ISSUED
()

void
IS\_FORMAT\_OF
()

void
IS\_PART\_OF
()

void
IS\_REFERENCED\_BY
()

void
IS\_REPLACED\_BY
()

void
IS\_REQUIRED\_BY
()

void
IS\_VERSION\_OF
()

void
LANGUAGE
()

void
LCC
()

void
LCSH
()

void
LICENSE
()

void
MEDIATOR
()

void
MEDIUM
()

void
MESH
()

void
MODIFIED
()

void
MOVINGIMAGE
()

void
PERIOD
()

void
PHYSICALOBJECT
()

void
POINT
()

void
PUBLISHER
()

void
REFERENCES
()

void
REPLACES
()

void
REQUIRES
()

void
RFC1766
()

void
RFC3066
()

void
RIGHTS
()

void
RIGHTS\_HOLDER
()

void
SERVICE
()

void
SOFTWARE
()

void
SOUND
()

void
SOURCE
()

void
SPATIAL
()

void
STILLIMAGE
()

void
SUBJECT
()

void
TABLE\_OF\_CONTENTS
()

void
TEMPORAL
()

void
TEXT
()

void
TGN
()

void
TITLE
()

void
TYPE
()

void
UDC
()

void
URI
()

void
VALID
()

void
W3CDTF
()

Methods

Description |
Methods (details)

ABSTRACT\_ (line 110)

void

ABSTRACT\_
()

ACCESS\_RIGHTS (line 116)

void

ACCESS\_RIGHTS
()

ALTERNATIVE (line 122)

void

ALTERNATIVE
()

AUDIENCE (line 128)

void

AUDIENCE
()

AVAILABLE (line 134)

void

AVAILABLE
()

BIBLIOGRAPHIC\_CITATION (line 140)

void

BIBLIOGRAPHIC\_CITATION
()

BOX (line 322)

void

BOX
()

COLLECTION (line 420)

void

COLLECTION
()

CONFORMS\_TO (line 146)

void

CONFORMS\_TO
()

CONTRIBUTOR (line 25)

void

CONTRIBUTOR
()

COVERAGE (line 31)

void

COVERAGE
()

CREATED (line 152)

void

CREATED
()

CREATOR (line 37)

void

CREATOR
()

DATASET (line 426)

void

DATASET
()

DATE (line 43)

void

DATE
()

DATE\_ACCEPTED (line 158)

void

DATE\_ACCEPTED
()

DATE\_COPYRIGHTED (line 164)

void

DATE\_COPYRIGHTED
()

DATE\_SUBMITTED (line 170)

void

DATE\_SUBMITTED
()

DCMI\_TYPE (line 328)

void

DCMI\_TYPE
()

DESCRIPTION (line 49)

void

DESCRIPTION
()

EDUCATION\_LEVEL (line 176)

void

EDUCATION\_LEVEL
()

EVENT (line 432)

void

EVENT
()

EXTENT (line 182)

void

EXTENT
()

FORMAT (line 55)

void

FORMAT
()

HAS\_FORMAT (line 188)

void

HAS\_FORMAT
()

HAS\_PART (line 194)

void

HAS\_PART
()

HAS\_VERSION (line 200)

void

HAS\_VERSION
()

IDENTIFIER (line 61)

void

IDENTIFIER
()

IMAGE (line 438)

void

IMAGE
()

IMT (line 334)

void

IMT
()

INTERACTIVE\_RESOURCE (line 444)

void

INTERACTIVE\_RESOURCE
()

ISO639\_2 (line 346)

void

ISO639\_2
()

ISO3166 (line 340)

void

ISO3166
()

ISSUED (line 236)

void

ISSUED
()

IS\_FORMAT\_OF (line 206)

void

IS\_FORMAT\_OF
()

IS\_PART\_OF (line 212)

void

IS\_PART\_OF
()

IS\_REFERENCED\_BY (line 218)

void

IS\_REFERENCED\_BY
()

IS\_REPLACED\_BY (line 224)

void

IS\_REPLACED\_BY
()

IS\_REQUIRED\_BY (line 230)

void

IS\_REQUIRED\_BY
()

IS\_VERSION\_OF (line 242)

void

IS\_VERSION\_OF
()

LANGUAGE (line 67)

void

LANGUAGE
()

LCC (line 352)

void

LCC
()

LCSH (line 358)

void

LCSH
()

LICENSE (line 248)

void

LICENSE
()

MEDIATOR (line 254)

void

MEDIATOR
()

MEDIUM (line 260)

void

MEDIUM
()

MESH (line 364)

void

MESH
()

MODIFIED (line 266)

void

MODIFIED
()

MOVINGIMAGE (line 450)

void

MOVINGIMAGE
()

PERIOD (line 370)

void

PERIOD
()

PHYSICALOBJECT (line 456)

void

PHYSICALOBJECT
()

POINT (line 376)

void

POINT
()

PUBLISHER (line 73)

void

PUBLISHER
()

REFERENCES (line 272)

void

REFERENCES
()

REPLACES (line 278)

void

REPLACES
()

REQUIRES (line 284)

void

REQUIRES
()

RFC1766 (line 382)

void

RFC1766
()

RFC3066 (line 388)

void

RFC3066
()

RIGHTS (line 79)

void

RIGHTS
()

RIGHTS\_HOLDER (line 290)

void

RIGHTS\_HOLDER
()

SERVICE (line 462)

void

SERVICE
()

SOFTWARE (line 468)

void

SOFTWARE
()

SOUND (line 474)

void

SOUND
()

SOURCE (line 85)

void

SOURCE
()

SPATIAL (line 296)

void

SPATIAL
()

STILLIMAGE (line 480)

void

STILLIMAGE
()

SUBJECT (line 91)

void

SUBJECT
()

TABLE\_OF\_CONTENTS (line 302)

void

TABLE\_OF\_CONTENTS
()

TEMPORAL (line 308)

void

TEMPORAL
()

TEXT (line 486)

void

TEXT
()

TGN (line 394)

void

TGN
()

TITLE (line 97)

void

TITLE
()

TYPE (line 103)

void

TYPE
()

UDC (line 400)

void

UDC
()

URI (line 406)

void

URI
()

VALID (line 314)

void

VALID
()

W3CDTF (line 412)

void

W3CDTF
()

Documentation generated on Fri, 17 Dec 2004 16:14:48 +0100 by phpDocumentor 1.3.0RC3
